# Validating midwifery professionals’ scope of practice and competency: A multi-country study comparing national data to international standards

**DOI:** 10.1371/journal.pone.0286310

**Published:** 2023-05-25

**Authors:** Suchandrima Chakraborty, Niranjan Saggurti, Richard Adanu, Delia A. B. Bandoh, Mabel Berrueta, Jewel Gausman, Ernest Kenu, Nizamuddin Khan, Ana Langer, Carolina Nigri, Magdalene A. Odikro, Veronica Pingray, Sowmya Ramesh, Paula Vázquez, Caitlin R. Williams, Charlotte E. Warren, R. Rima Jolivet

**Affiliations:** 1 Population Council, New Delhi, India; 2 Department of Population, Family, and Reproductive Health, University of Ghana School of Public Health, Accra, Ghana; 3 Department of Epidemiology and Disease Control, University of Ghana School of Public Health, Accra, Ghana; 4 Institute for Clinical Effectiveness and Health Policy (Instituto de Efectividad Clínica y Sanitaria (IECS), Buenos Aires, Argentina; 5 Women and Health Initiative, Department of Global Health and Population, Harvard University T.H. Chan School of Public Health, Boston, Massachusetts, United States of America; 6 Department of Health Science, School of Kinesiology and Physiatry, University of La Matanza, Province of Buenos Aires, Argentina; 7 Department of Maternal & Child Health, Gillings School of Global Public Health, University of North Carolina at Chapel Hill, Chapel Hill, North Carolina, United States of America; 8 Social and Behavioral Science Research, Population Council, Washington, DC, United States of America; McGill University, CANADA

## Abstract

**Background:**

There is a global shortage of midwives, whose services are essential to meet the healthcare needs of pregnant women and newborns. Evidence suggests that if enough midwives, trained and regulated to global standards, were deployed worldwide, maternal, and perinatal mortality would decline significantly. Health workforce planning estimates the number of midwives needed to achieve population coverage of midwifery interventions. However, to provide a valid measure of midwifery care coverage, an indicator must consider not only the raw number of midwives, but also their scope and competency. The tasks midwives are authorized to deliver and their competency to perform essential skills and behaviors provide crucial information for understanding the availability of safe, high-quality midwifery services. Without reliable estimates for an adequate midwifery workforce, progress toward ending preventable maternal and perinatal mortality will continue to be uneven. The International Labor Organization (ILO) and the International Confederation of Midwives (ICM) suggest standards for midwifery scope of practice and competencies. This paper compares national midwifery regulations, scope, and competencies in three countries to the ILO and ICM standards to validate measures of midwife density. We also assess midwives’ self-reported skills/behaviors from the ICM competencies and their acquisition.

**Methods and findings:**

We compared midwives’ scope of practice in Argentina, Ghana, and India to the ILO Tasks and ICM Essential Competencies for Midwifery Practice. We compared midwives self-reported skills/behaviors with the ICM Competencies. Univariate and bivariate analysis was conducted to describe the association between midwives’ skills and selected characteristics. National scopes of practice matched two ILO tasks in Argentina, four in India, and all in Ghana. National standards partially reflected ICM skills in Categories 2, 3, and 4 (pre-pregnancy and antenatal care; care during labor and birth; and ongoing care of women and newborns, respectively) in Argentina (range 11% to 67%), mostly in India (range 74% to 100%) and completely in Ghana (100% match). 1,266 midwives surveyed reported considerable variation in competency for skills and behaviors across ICM Category 2, 3, and 4. Most midwives reported matching skills and behaviors around labor and childbirth (Category 2). Higher proportions of midwives reported gaining basic skills through in-service training and on-job-experience than in pre-service training.

**Conclusion:**

Estimating the density of midwives needed for an adequate midwifery workforce capable of providing effective population coverage is predicated on a valid numerator. A reliable and valid count of midwives to meet population needs assumes that each midwife counted has the authority to exercise the same behaviors and reflects the ability to perform them with comparable competency. Our results demonstrate variation in midwifery scopes of practice and self-reported competencies in comparison to global standards that pose a threat to the reliability and validity of the numerator in measures of midwife density, and suggest the potential for expanded authorization and improved education and training to meet global reference standards for midwifery practice has not been fully realized. Although the universally recognized standard, this study demonstrates that the complex, composite descriptions of skills and behaviors in the ICM competencies make them difficult to use as benchmark measures with any precision, as they are not defined or structured to serve as valid measures for assessing workforce competency. A simplified, content-validated measurement system is needed to facilitate evaluation of the competency of the midwifery workforce.

## Introduction

Despite focused attention and investment over the last two decades to improve maternal and newborn health (MNH), today there is still an estimated global shortage of 900,000 midwives, whose services are essential to meet the healthcare needs of pregnant women and their newborns [1]. Evidence suggests that when enough midwives are trained and regulated to global standards and deployed within an enabling environment, maternal and perinatal mortality go down [2]. It is estimated that 2.2 million maternal and newborn deaths could be averted (41% maternal deaths, 39% neonatal deaths, and 26% stillbirths) by 2035 if coverage of midwife-led care interventions was increased by 25% every 5 years [2]. Achieving population coverage of essential services that are within the global standard scope of practice for midwives is a critical strategy for improving both quality MNH care and coverage [3]. However, progress in implementing midwifery practice regulations that allow midwives to deliver the full range of MNH care interventions recognized in global standards has not been uniform [4–6]. In 2019, guiding organizations including UNFPA, WHO, and ICM issued a call to action for an enabling environment for midwives, and stated that given strong “evidence that midwives, educated to ICM standards, licensed, regulated … and practicing within an enabling environment will provide high quality care that transforms maternal and newborn health outcomes”, all midwives should have the ability to work to their full scope of practice [7]. These assumptions underpin the targets and estimates for the number of midwives needed to scale up midwifery to achieve the desired maternal and newborn health benefits.

Health workforce planners estimate the number of workers needed to achieve population coverage of essential interventions. Without clear, comparable, measurable standards for midwifery practice that policymakers, programmers, and frontline midwifery professionals can implement across countries, national decision makers lack the basic information needed to estimate and ensure a sufficient number of midwives to deliver essential quality MNH care coverage; without reliable estimates for an adequate midwifery workforce, progress toward ending preventable maternal and perinatal mortality will continue to be uneven [8].

Global standards for midwifery practice are described by the International Labor Organization (ILO) and the International Confederation of Midwifery (ICM). The International Standard Classification of Occupations 2008 (ISCO-08), describes the scope of practice for midwifery professionals and associate midwifery professionals based on job tasks with varying levels of responsibility [9], and performed within their professional occupations, rather than on professional designation as “midwives” per se [9].

The ICM developed core competencies for basic midwifery practice [10], referenced in the following definition, “*A midwife is a person who has successfully completed a midwifery education programme that is based on the ICM Essential Competencies for Basic Midwifery Practice and the framework of the ICM Global Standards for Midwifery Education and is recognized in the country where it is located; who has acquired the requisite qualifications to be registered and/or legally licensed to practice midwifery and use the title ‘midwife’; and who demonstrates competency in the practice of midwifery”* [11–13]. Together, the ILO’s ISCO-8 and the ICM Competencies set global standards for midwifery practice, by defining both the scope (job tasks) and core competencies (knowledge, skills, and behaviors) that serve as benchmarks for basic midwifery practice [9, 11, 14]. The estimated number of deaths averted if midwifery care were scaled up is predicated on the assumption that those midwives are trained and regulated to such global standards [2]. However, little is known about the uptake of these global standards within national policies and guidelines, or how well the implementation of these policies is reflected at country level.

The 2015 “Strategies toward Ending Preventable Maternal Mortality (EPMM)” report [15] outlines strategies for reducing maternal mortality by 2030 and maternal mortality targets to measure progress globally. In 2017, a menu of indicators was proposed that could be used to by national planners to monitor critical upstream drivers of maternal mortality, tailored to the EPMM Strategies 11 Key Themes [16]. Monitoring the density and distribution of midwives was prioritized to drive progress toward both health system strengthening and health equity [15]. Two differently defined indicators for calculating the number of midwives that would be sufficient for coverage of the population in need of midwifery care were suggested, each one taking a different approach to construct the measure [16]. The optimal operationalization is unknown.

The first, Sustainable Development Goal 3.c.1. (health worker density and distribution), focuses on density of the healthcare workforce per 10,000 population [17], and describes the number of health workers, disaggregated by nurses and midwives (numerator), per the total population (denominator). The second proposed indicator, “density of midwives per births per district” focuses specifically on midwives, redefines the population in need of midwifery services, and adds a measure of geographic distribution. Both measures are subject to potential threats to validity of both the numerator and denominator; however, this study focused on the numerator, the number of midwifery professionals available and able to deliver quality maternal newborn care (QMNC) per the best available reference standards. Density and distribution of midwives to meet population needs in countries across the world is a critical health system-level measure whose validity has not been systematically assessed [18].

For an indicator to provide a valid measure of the effective coverage of midwifery care [19], it must consider not only the raw numbers of midwives, but also the scope and competency of the midwifery workforce. The range of tasks that midwives are authorized to deliver and their competency to perform essential skills and behaviors provide crucial information for understanding the availability of safe, high-quality midwifery services. Accounting for the scope of practice and competency of midwives in the numerator of indicators used to estimate the density of midwives is important given cross-national variation in midwifery education, training and regulation, resulting in a lack of uniformity in the care that midwives can provide [6]. A valid estimate of the number of midwives needed implies that each midwife counted reflects an equal measure of competency and potential scope of practice. The validity of an indicator to estimate the number of midwives sufficient to provide QMNC commensurate with national needs depends on a reliable estimate of the number of midwives who also demonstrate the essential competencies and are empowered to deliver full-scope midwifery care.

MNH policy indicators are seldom systematically validated [20] and this is true for maternity workforce measures as well, with the recent exception of validation of the skilled birth attendant indicator in 2016 [21]. This study is part of a larger set of research studies that aim to assess the validity of key policy and health system level indicators from the monitoring framework for the EPMM Strategies. Key stakeholders in each of our research settings prioritized midwifery density as a key indicator for national monitoring and affirmed that research to assess its validity would be useful for policy and planning. In the technical approach to assessing measure validity, criterion-related validity seeks to test the empirical association of a measure with a reference standard. To date, there is no evidence to explore the agreement between country regulations and global standards governing midwifery professionals’ tasks and skills. Such information is needed to strengthen measures of the availability of an adequate midwifery workforce. The extent to which national regulatory frameworks are aligned with global standards can increase confidence in the reliability of the measure of an adequate midwifery workforce. Verifying whether global policy standards for essential midwifery competencies are aligned with empirical data evaluating those same competencies at country level can provide further evidence to assess the validity of estimates of the density of midwives.

We sought to identify the most universally applicable and universally recognized reference standards for midwifery professional tasks and essential competencies for basic midwifery practice. and thus chose the ILO and ICM reference standards. We compared legal practice acts and regulatory frameworks that define the midwifery scope of work and essential competencies (tasks, skills, and behaviors) at country-level with the global benchmarks set by ILO and ICM. In addition, we investigated the extent to which midwives reported skills and behaviors that matched the ICM competencies. The research, conducted in Argentina, Ghana, and India, explores the following questions:

1. How do the regulatory documents that define scope of practice for midwives and associate midwifery professionals in each country, compare to the global benchmarks from the International Labor Organization and the International Confederation of Midwives?
2. How do self-reported skills and behaviors for midwives and midwifery associate professionals compare to those outlined in the ICM core competencies for the practice of midwifery?
3. Is self-reported possession of essential midwifery competencies associated with any individual or contextual characteristics of respondents?
4. Where do midwives report gaining essential competencies: pre-service training, in-service training or ‘on the job training’ (OJT)?

## Methods

### Study settings

This study is part of a larger research project for which three study settings were selected purposively to represent a geographically diverse set of low- and middle-income countries with a significant burden of maternal mortality and based on local stakeholder interest and research capacity. In each country, four subnational districts or provinces were selected based on an index of key maternal health metrics designed to reflect health system performance. Study sites were selected through a multi-stage sampling approach and include four subnational areas in Argentina (Buenos Aires, Jujuy, La Pampa, and Salta provinces), Ghana (Techiman North, Sunyani Municipal, Bunkpurugu Yunyoo, and Tolon districts), and India (Gonda, Meerut, Krishnagiri and Thiruvallur districts). First, a state or region in the top and bottom quartiles were selected and within each of them, a district or province in the top and bottom quartile was selected (terciles were used in Argentina due to low population density). More information on selection of the study settings is available in the published study protocol [18].

### Study design

This observational study explores multiple data sources to assess the validity of the numerator of policy-level midwifery workforce indicators used at global and national level. These indicators were prioritized for validation research through consultation with key national stakeholders including official government representatives in each country. (In India, a new government initiative to educate a new cadre of advance-practice independent nurse practitioner midwives contributed to specific interest in this indicator.) We performed a desk review of policy, legal and regulatory data in three countries. We also conducted a cross-sectional primary survey with all eligible health providers–those whose job descriptions matched the ISCO-8 occupational classification for midwifery professionals or associate midwifery professionals (henceforth described collectively as midwives)–employed in all eligible facilities in each district/province.

#### Mapping and analysis of national policies and guidelines

To address the first validation question, we conducted a comprehensive desk review of national and subnational laws, regulations, health workforce guidelines and polices in Argentina, Ghana, and India. This included a systematic search of national websites and repositories using keywords related to midwifery regulation and scope of practice; consultations with midwifery experts including those from Ministries of Health and national midwifery institutions; and a hand search of any relevant hard copies from government and nursing and midwifery institutions.

National policies and guidelines were defined as any document that provided guidance on the scope of practice for midwifery professionals on record, defined according to the ILO classification of occupations as ‘*planning*, *providing*, *and evaluating care and support services for women and babies before*, *during and after pregnancy and managing complications*.’ The initial search for documents at national level took place between May-July 2020 in all three study countries.

In each country, a standard data extraction form was used to capture and compare data from the source documents to the tasks in the ILO classification system [9] (Box 1), and the ‘skills and behaviors’ listed for the ICM Competencies, version released January 2019 [13] (Box 2). (Note: a subsequent update appeared in October 2019, after development and cognitive testing of our study instruments had been completed.) Two team members in each country compared all national policy and regulation documents relevant to midwifery skills with the ILO classification and ICM competencies and independently coded each document. Any discrepancies were resolved through discussion. A third team member was brought in as needed to reach consensus.

**Box 1. Tasks for each ILO- International Standard Classification of Occupations.**

**Table:** 

Description	Task Includes
Midwifery professionals plan, manage, provide and evaluate midwifery care services before, during and after pregnancy and childbirth. They provide delivery care for reducing health risks to women and newborn children, working autonomously or in teams with other health care providers.	A. Planning, providing and evaluating care and support services for women and babies before, during and after pregnancy and childbirth according to the practice and standards of modern midwifery care; B. Providing advice to women and families and conducting community education on health, nutrition, hygiene, exercise, birth and emergency plans, breastfeeding, infant care, family planning and contraception, lifestyle and other topics related to pregnancy and childbirth; C. Assessing progress during pregnancy and childbirth, managing complications and recognizing warning signs requiring referral to a medical doctor with specialized skills in obstetrics; D. Monitoring the health status of newborns, managing complications and recognizing warning signs requiring referral to a medical doctor with specialized skills neonatology; E. Monitoring pain and discomfort experienced by women during labour and delivery and alleviating pain using a variety of therapies, including the use of painkilling drugs; F. Reporting births to government authorities to meet legal and professional requirements; G. Conducting research on midwifery practices and procedures and disseminating findings such as through scientific papers and reports; H. Planning and conducting midwifery education activities in clinical and community settings.
Midwifery associate professionals provide basic health care and advice before, during and after pregnancy and childbirth. They implement care, treatment and referral plans usually established by medical, midwifery and other health professionals.	A. Providing advice to women, families and communities on health, nutrition, hygiene, exercise, birth and emergency plans, breastfeeding, infant care, family planning and contraception, lifestyle and other topics related to pregnancy and childbirth; B. Assessing progress during pregnancy and childbirth, and recognizing signs and symptoms requiring referral to a health professional; C. Providing delivery care, usually only in absence of identified potential complications, or assisting medical doctors or midwifery professionals with delivery care; D. Providing care and support to women and newborns following childbirth, monitoring their health status, and identifying signs and symptoms requiring referral to a health professional.

**Box 2. Skills and behaviors for each ICM competency.**

**Table:** 

**Skills**	**Sub domains**
General Competency: Competencies in this category are about the midwife’s autonomy and accountabilities as a health professional, the relationships with women and other care providers, and care activities that apply to all aspects of midwifery practice. **(Category 1)**	1a. Assume responsibility for own decisions and actions as an autonomous practitioner 1b. Assume responsibility for self-care and self-development as a midwife 1c. Appropriately delegate aspects of care and provide supervision 1d. Use research to inform practice 1e. Uphold fundamental human rights of individuals when providing midwifery care 1f. Adhere to jurisdictional laws, regulatory requirements, and codes of conduct for midwifery practice 1i. Facilitate normal birth processes in institutional and community settings, including women’s homes 1j. Assess the health status, screen for health risks, and promote general health and well-being of women and infants 1k. Prevent and treat common health problems related to reproduction and early life 1l. Recognize abnormalities and complications and institute appropriate treatment and referral 1m. Care for women who experience physical and sexual violence and abuse
Skills necessary to provide pre-pregnancy and antenatal care **(Category 2)**	2a. Provide pre-pregnancy and antenatal care 2b. Determining health status of woman, 2c. assessing fetal well-being, 2d. monitoring the progression of pregnancy, 2e. promoting and supporting health behaviors that improve well-being, 2f. providing anticipatory guidance related to pregnancy, birth, breastfeeding, parenthood, and change in the family, 2g. detecting, stabilizing, managing, and referring women with complicated pregnancies, 2h. assisting the woman and her family to plan for an appropriate place of birth, 2i. providing care to women with unintended or mistimed pregnancy.
Skills necessary to provide care during labor & birth **(Category 3)**	3a. Promoting physiologic labour and birth, 3b. managing a safe spontaneous vaginal birth and preventing complications, 3c. providing care of the newborn immediately after birth
Skills necessary to provide ongoing care of women and newborns **(Category 4)**	4a. Providing postnatal care for the healthy woman, 4b. providing care to healthy newborn infant, 4c. promoting and supporting breastfeeding, 4d. detecting, treating, and stabilizing postnatal complications in woman and referring as necessary, 4e. detecting, stabilizing, and managing health problems in newborn infant and refer, if necessary, 4f. providing family planning services.

#### Midwifery surveys and analysis

For the second validation question, we surveyed midwifery professionals from all eligible health facilities in the selected provinces/districts of each study setting. For each selected district/province, all public health facilities providing any of thirteen maternal health-related services were eligible to be included in the study. In Ghana, both public and private registered hospitals were included. A list of all such facilities was obtained from the local health department in each study area. The thirteen maternal health-related services are enumerated in the WHO Maternal Newborn Child and Adolescent Health (MNCAH) Policy Survey [22] as essential services that should be freely available to all women of reproductive age in every country. They include family planning, antenatal care (ANC) and insecticide-treated nets, management of normal and complicated childbirth, pregnancy and childbirth-related diagnostic testing and treatment, postnatal care, testing and treatment for sexually transmitted infections, testing and treatment for syphilis and HIV, and screening for cervical cancer.

Facility selection differed somewhat by country. In Argentina, facilities offering all categories of services provided by midwives were first identified, and those that did not offer abortion services or employ midwives were then excluded for feasibility reasons. From the resulting list, a purposive sample of facilities at all three levels (primary, secondary, and tertiary) was selected. In Ghana, all eligible primary and secondary health facilities in the four study districts that provided birth-care and/or other maternal health-related services as per the above WHO MNCAH Policy Survey were selected (24). In India, all eligible secondary- and tertiary-level facilities were included, as well as a random sample of 20 eligible primary health care facilities in each study area due to the large number of primary care sites.

### Study participants

Each health institution’s human resource or payroll department provided a list of midwifery professionals currently employed in government health facilities (for Argentina, Ghana, and India) and registered private institutions (for Ghana). Participants were considered eligible for interview if they were old enough to provide consent (18 years or older in Ghana and India, 16 years or older in Argentina), and confirmed during recruitment that they were actively engaged in ‘*planning*, *providing*, *and evaluating care and support services for women and babies before*, *during and after pregnancy and managing complications*’ in their current job regardless of their title, as per the ILO occupational classification system for a midwifery professional or midwifery associate professional. All eligible health professionals from each study district were invited for interview. The mode of data collection (between May and December 2020) was, determined by in-country guidance regarding the COVID-19 pandemic. In-person interviews were conducted in Ghana, telephone interviews were employed in India, and electronic email surveys were used in Argentina.

Trained data collectors used a standardized, structured survey questionnaire in all settings. Questions related to respondents’ socio-demographic background, their self-reported level of skill for each competency; locus of training to achieve each competency; frequency and recency of the performance of tasks related to each competency; and the reasons for non-performance of specified tasks in their current job. Questionnaires were translated into local languages, and cognitive testing to pilot the survey questions was conducted in all three countries to ensure consistency in the meaning after translation.

**Fig 1:**
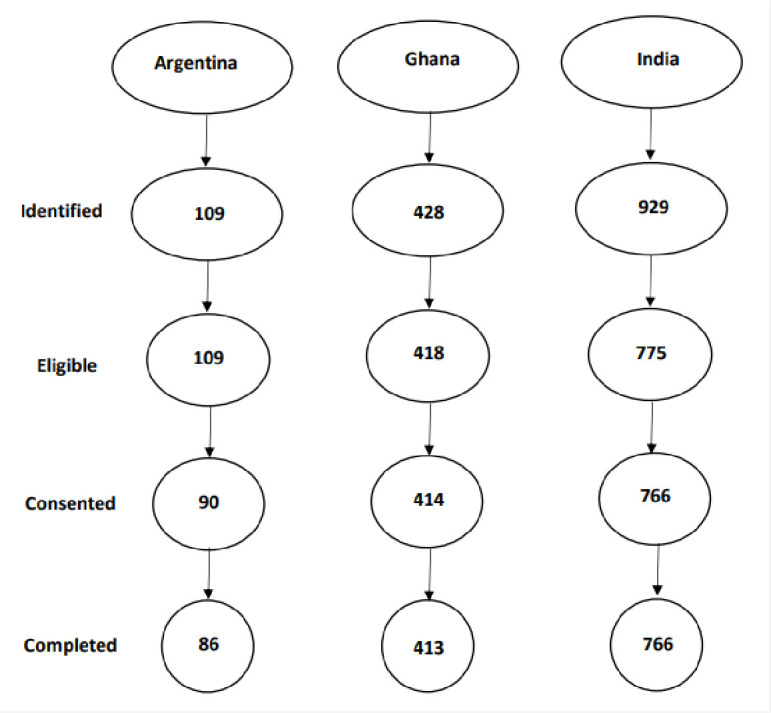
Flowchart of participant selection.

The response rate among eligible midwives who consented and completed interviews varied (Fig 1). The reasons for non-response and non-completion of interview were attributed to the effects of COVID-19, as some midwives declined participation due to excessive demands under COVID-19, some were on extended sick leave, and some were unresponsive after multiple (three) attempts to reach them or reminders.

### Measures

We asked participants to report whether they possess the skills and behaviors listed for ICM Competencies in Categories 2, 3, & 4 (see Box 2), which focus specifically on pre-pregnancy and antenatal care, care during labour and birth, and ongoing care of women and newborns. We chose not to include Category 1 of the ICM Competencies, “General Competencies”, in the survey due to the more abstract, less objective nature of the skills and behaviors included under this Category (see Box 2).

Within each category, respondents were asked to self-report whether they believe they possess each skill/behavior described for all included competencies. For example, midwives were asked “Do you have the necessary skills to confirm pregnancy and estimate gestational age from history, physical exam, laboratory test and/or ultrasound?”, for the competency of determining health status of women in the pre-pregnancy and antenatal care category. Responses were captured using the following options: *none*, *some*, *most*, *all*, and *refused to answer*. All participants who reported possessing any of the skills/behaviors under a specific task were asked a follow-up question, “How did you gain the skills?” The response options for this question were: “pre-service education/school”, “in-service/continuing education”, and “on the job experience”. Respondents could choose more than one response.

Further, two stratification variables were chosen to analyze the tasks for each competency category: number of years of service, and type of facility. The number of years of service were coded as: <5 years, 5–10 years, >10 years; and the type of facility was coded as: primary, secondary, and tertiary health facilities.

### Analysis

For the secondary data, we calculated the percent agreement between the description of the tasks, skills, and behaviors that represent the scope of practice for midwives and midwifery professionals in the international standards (ILO and ICM) and the national laws and regulations. We assigned a score of one for each component of the scope of practice from the international standard-setting documents that was reflected in the national source documents, with 50% of the score attributed if the component was mentioned in general, and up to an additional 50% of the score for mention of specific tasks/responsibilities detailed in the international standard for each component. We then calculated the percent agreement between the ILO tasks and ICM competencies, and the country-specific national laws and regulations, for each national source document.

For the primary data, we analyzed midwives’ self-reported skills and behaviors and their actual performance of those skills and behaviors over the previous 90-day period prior to the interview in each country. Univariate analysis was used to calculate the percentage of midwives who reported having ALL the skills related to each ICM competency, how they obtained those skills, and their performance of skills in the past 90 days. A composite measure summarizing skills listed under each competency sub-category (i.e., 2a-2i, 3a-3c, and 4a-4f) was also calculated based on the reported skills of the respondents. Furthermore, we performed descriptive statistics and assessed differences in practice by district, facility type, and professional designation, i.e., midwifery and associate midwifery roles. Stata version 16.0 was used for all statistical analyses.

### Ethical considerations

The study was approved by the ethical review board in the Office of Human Research Administration at Harvard University (IRB19-1086). Each country also received approval for the study through local institutional ethical review boards. For Argentina, local institutional review boards approved the study (Comité de Ética de la Investigación de la Provincia de Jujuy–Approval ID Not applicable. Comisión Provincial de Investigaciones Biomédicas de la Provincia de Salta–Approval ID 321-284616/2019. Consejo Provincial de Bioética de la Provincia de La Pampa–Approval ID Not applicable. Comité de Ética Central de la Provincia de Buenos Aires–Approval ID 2919-2056-2019). The Ghana Health Service Ethics Review Committee (GHS-ERC022/08/19) approved the study in Ghana, and in India, the local institutional review board Sigma-IRB (IRB Number: 10052/IRB/19-20) approved the study.

Informed consent procedures were submitted and approved by all ethical review boards in each setting. Written informed consent was obtained from all participants prior to data collection. Data collectors explained the study purpose and procedures and the voluntary nature of the research. Potential participants were encouraged to ask questions and any concerns were addressed by the research investigators, with emphasis on participant’s ability to withdraw at any time. Electronic informed consent was used in Argentina. Precautions were taken to secure and de-identify data, and data protection procedures. Throughout the data collection process, data entry and analysis, the anonymity and confidentiality of the data was carefully maintained. To reduce the risk of deductive disclosure, data was aggregated in a manner that individual province/district, facility, or participant could not be potentially identified.

## Results

### Agreement between national scopes of practice for midwifery professionals and the ILO classification of occupations task list

Argentina has two national standard-setting documents that govern midwifery practice, Ghana has three, and India has four. Agreement between these national regulatory documents and the global standards is presented in Tables 1 and 2.

**Table 1:** Percent agreement between national scopes of practice for midwifery professionals and the ILO standard classification of occupations task list.

	Weighted Score* (% agreement)							
	Task A	Task B	Task C	Task D	Task E	Task F	Task G	Task H
**Argentina (Midwifery professional)**								
National Record 1 (Ley 17.132)	50.0	0.0	100.0	0.0	0.0	100.0	0.0	0.0
National Record 2 (Decreto Reglemenentario de la Ley 17.132)	50.0	0.0	100.0	0.0	0.0	100.0	0.0	0.0
**Ghana (Midwifery professional)**								
National Reproductive Health Policy and Standards	100.0	100.0	100.0	100.0	100.0	100.0	100.0	100.0
National Safe Motherhood Protocol–Second Edition	0.0	0.0	0.0	0.0	0.0	0.0	0.0	0.0
Ghana Health Service Job Description for Midwives	0.0	0.0	0.0	0.0	0.0	0.0	0.0	0.0
**India (Midwifery professional)**								
Guidelines for ANC and SBA by ANMs/LHVs/SNs	33.0	75.0	100.0	100.0	100.0	100.0	0.0	0.0
A Handbook for ANMs/LHVs/SNs	33.0	50.0	100.0	100.0	50.0	100.0	0.0	0.0
Operational Guidelines on MNH	33.0	75.0	100.0	100.0	0.0	0.0	0.0	0.0
Syllabus and Regulations of Nurses (INC)	33.0	75.0	100.0	100.0	100.0	100.0	0.0	100.0
**India (Midwifery associate professional) **								
Guidelines for ANC and SBA by ANMs/LHVs/SNs	93.0	100.0	100.0	50.0	NA	NA	NA	NA
A Handbook for ANMs/LHVs/SNs	50.0	100.0	100.0	50.0	NA	NA	NA	NA
Operational Guidelines on MNH	68.0	100.0	100.0	50.0	NA	NA	NA	NA
Syllabus and Regulations of Nurses (INC)	0.0	50.0	100.0	50.0	NA	NA	NA	NA

*All tasks were weighted equally during final calculation *For details of tasks, refer to Box 1

NA: Not Applicable; ANC: Antenatal Care; SBA: Skilled Birth Attendant; ANM: Auxiliary Nurse Midwife; LHV: Lady Health Visitor; SN: Staff Nurse; MNH: Maternal and Newborn Health

**Midwifery professional**–Task A: Planning, providing and evaluating care and support services; Task B: Providing advice to women and families and conducting community education; Task C: Assessing progress during pregnancy and childbirth, managing complications and recognizing warning signs; Task D: Monitoring the health status of newborns, managing complications and recognizing warning signs; Task E: Monitoring pain and discomfort experienced by women during labour and delivery and alleviating pain; Task F: Reporting births; Task G: Conducting research; Task H: Conducting midwifery education activities**. Midwifery associate professional—**Task A: Providing advice; Task B: Assessing progress during pregnancy and childbirth; Task C: Providing delivery care; Task D: Providing care and support to women and newborns following childbirth

**Table 2:** Percent agreement between national midwifery scopes of practice and ICM Competencies.

	Weighted Score* (% agreement)			
	Category 1	Category 2	Category 3	Category 4
**Argentina**				
National Record 1 (Ley 17.132)	14.0	11.0	67.0	17.0
National Record 2 (Decreto Reglemenentario de la Ley 17.132)	14.0	22.0	67.0	33.0
**Ghana**				
National Reproductive Health Policy and Standards	45.0	100.0	100.0	100.0
National Safe Motherhood Protocol–Second Edition	37.0	100.0	100.0	100.0
Ghana Health Service Job Description for Midwives	92.0	100.0	100.0	100.0
**India**				
Guidelines for ANC and SBA by ANMs/LHVs/SNs	45.0	74.0	100.0	100.0
A Handbook for ANMs/LHVs/SNs	27.0	81.0	100.0	92.0
Operational Guidelines on MNH	54.0	81.0	100.0	92.0
Syllabus and Regulations of Nurses (INC)	23.0	92.0	100.0	83.0

*All tasks were weighted equally during final calculation

* For details of Competencies please refer to Box 2

NA: Not Applicable; ANC: Antenatal Care; SBA: Skilled Birth Attendant; ANM: Auxiliary Nurse Midwife; LHV: Lady Health Visitor; SN: Staff Nurse; MNH: Maternal and Newborn Health

Category 2: Skills necessary to provide pre-pregnancy and antenatal care; Category 3: Skills necessary to provide care during labor & birth; Category: Skills necessary to provide ongoing care of women and newborns.

The comparison between national scopes of practice for midwives and the ILO classification of occupations for all eight tasks (Tasks A–H described in Box 1) is presented in Table 1. Only two tasks from the ILO global standard classification system for midwifery professionals were represented in all three countries’ national guidelines (C and F). All eight ILO tasks were listed in national documents in Ghana, four in India and two in Argentina. .)”.

### Agreement between national scopes of practice for midwifery professionals and the ICM skills in competencies 2–4

The comparison between national scope of practice documents for midwives and the ICM Competencies Skills and Behaviors, Categories 2–4, is presented in Table 2. In Argentina, both national source documents only partially reflected the skills included in the ICM Competencies in Categories 2–4. The Ghana Health Service national source documents aligned completely with the ICM skills in Categories 2, 3 and 4. In India, the ICM skills in Category 3 were reflected fully within all four national policy documents.

### Midwives’ self-assessment of skills/behaviors for ICM competencies

A total of 1,266 midwives responded to the survey that asked them to report their skills reflected in the ICM Competencies in Category 2 (pre-pregnancy and antenatal care), Category 3 (care during labor and birth), and Category 4 (ongoing care of women and newborns). **Table 3** describes the characteristics of the respondents (average age, gender, facility level and hours worked per week).

**Table 3:** Survey respondent characteristics.

	Argentina	Ghana	India	
Total number of participants (n)	86	414	500*	266**
Mean age (SD)	40.6 (8.0)	34.2 (8.9)	36.0 (8.6)	36.4 (10.9)
Gender % (n)				
Male	7.0 (6)	14.0 (58)	1.4 (7)	6.77 (18)
Female	93.0 (80)	86.0 (356)	98.6 (493)	93.2 (248)
Mean years in service (SD)	13.5 (8.4)	7.4 (7.6)	10.3 (7.9)	10.1 (10.0)
**Facility type % (n)**				
Primary Care	44.2 (38)	84.3 (349)	64.9 (135)	35.1 (73)
Secondary Care	31.4 (27)	17.5 (65)	63.2 (172)	36.8 (100)
Tertiary Care	73.3 (63)	—	67.5 (193)	32.5 (93)
**Number of hours worked per week**				
>40 hours per week	54.7 (47)	70.1 (290)	60.7 (283)	39.3 (183)
40 hours per week	29.1 (25)	17.6 (73)	12.5 (7)	87.5 (49)
<40 hours per week	16.3 (14)	10.6 (44)	86.1 (210)	13.9 (34)
Refused	-	1.7 (7)	-	-

Note: NA: Not Applicable; CHC/PHC—Community Health Centre/Primary Health Center

* Nurse-midwives

** Associate nurse midwives

**Table 4** details the percentage of midwives interviewed who reported possessing the skills and behaviors included in ICM Competencies, Category 2 (pre-pregnancy and antenatal care), Category 3 (intrapartum care 3) and Category 4 (ongoing care for women and newborns). Further detail is available in **S1–S3 Tables**.

**Table 4:** Midwives who reported possessing the skills and behaviors included in the ICM competencies, categories 2–4, by country.

	Argentina n-86	Ghana n-414	India n-766
**Category 2 –Provide pre-pregnancy and antenatal care**	%	%	%
2.a Provide pre-pregnancy care	19.8	50.7	11.3
2.b Determine health status of woman	11.6	42.2	16.1
2.c Assess fetal well-being	62.8	51.9	16.3
2.d Monitor the progression of pregnancy	34.9	57.5	36.4
2.e Promote/support health behaviours that improve wellbeing	23.3	61.4	13.3
2.f Provide anticipatory guidance related to pregnancy, birth, breastfeeding, parenthood, and change in the family	38.4	58.5	14.0
2.g Detect, stabilise, manage, and refer women with complicated pregnancies	9.3	46.9	8.2
2.h Assist woman and her family to plan for an appropriate place of birth	36.1	59.9	15.9
2.i Provide care to women with unintended/mistimed pregnancy	10.5	45.2	7.4
**Category 3—Care during labour and birth**	%	%	%
3.a Promote physiologic labour and birth	29.1	37.7	4.7
3.b Manage a safe spontaneous vaginal birth and prevent complications	15.1	37.2	4.7
3.c Provide care of the newborn immediately after birth	7.0	65.7	31.1
**Category 4—Ongoing care of women and newborns**	%	%	%
4.a Provide postnatal care for the healthy woman	37.2	52.9	19.7
4.b Provide care to healthy newborn infant	2.3	63.0	26.2
4.c Promote and support breastfeeding	22.1	68.6	37.1
4.d Detect, treat, and stabilise postnatal complications in woman and refer as necessary	8.1	56.0	17.8
4.e Detect, stabilise, and manage health problems in newborn infant and refer if necessary	2.3	54.8	23.6
4.f Provide family planning services	38.4	62.3	41.2

The nine subcategories of competencies included within Category 2 are listed in **Table 4**. The percent of midwives from Argentina who reported possessing the skills and behaviors in these subcategories varied, with the highest number reporting they possessed the skills to *‘assess fetal well-being*’ (63%) ‘*provide anticipatory guidance related to pregnancy*, *birth*, *breastfeeding’* (38%). For some ICM skills and behaviors in Category 2, more than half of midwives in Ghana reported possessing them, specifically to ‘*assist woman and her family to plan for an appropriate place of birth’* (60%) and *‘promote and support healthy behaviors’* (61%). In India, for all sub-categories in Category 2, respondents reported skills ranged between 7% and 36%.

There are three sub-categories of ICM competencies included in Category 3. The percent of midwives who reported having the skills described in these sub-categories ranged from 7% to 29% in Argentina, 37% to 68% in Ghana, and 5% to 31% in India.

Within Category 4, there are six sub-categories. The highest percentages (top 3) of midwives in Argentina who reported skills and behaviors included in the ICM competencies for ongoing care for women and newborns ranged from 22–38% in Argentina, 62% to 69% in Ghana and 26% to 41% in India,) (see **Table 4**).

**Box 3** highlights the specific skills and behaviors that respondents most frequently reported, and **Box 4** highlights the least frequently reported skills and behaviors for the subcategories within the ICM Competencies, categories 2–4. For all three countries there is wide variation on reported skills and behaviors.

**Box 3. The five skills and behaviors most frequently reported for ICM competencies in categories 2–4, by country**

**Table:** 

**Argentina**					
**Category 2 (Pre-pregnancy and antenatal care)**		**Category 3 (Care during labor and birth)**		**Category 4 (Ongoing care for women and newborns)**	
**Skill or Behavior**	**%MWs**	**Skill or Behavior**	**%MWs**	**Skill or Behavior**	**%MWs**
Confirm pregnancy and determine gestational age; refer as needed	88%	Assess regularly parameters of maternal-fetal status, and e.g., vital signs, contractions, cervical changes, and fetal descent	88%	Conduct a focused physical exam to assess breast changes and involution	85%
Provide information about danger signs	87%	Deliver placenta and membranes, inspect for completeness	88%	Monitor blood loss and other body functions	84%
Assess fetal movements and ask woman about fetal activity	86%	Delay cord clamping	85%	Provide (FP) methods according to scope of practice and protocols, or refer to another provider	79%
Prepare the woman and family to recognize labour onset	84%	Obtain relevant obstetric and medical history	80%	Review history of pregnancy, labor, and birth	78%
Provide information about postpartum needs, including contraception, care of newborn infants, and the importance of exclusive breast feeding for infant health	79%	Provide skin-to -care and warm environment	80%	Provide information about safe sex, FP methods appropriate for the immediate postnatal period, and pregnancy spacing	78%
**Ghana**					
**Category 2 (Pre-pregnancy and ANC)**		**Category 3 Care during labor and birth)**		**Category 4 (Ongoing care for women and newborns)**	
**Skill or Behavior**	**%MWs**	**Skill or Behavior**	**%MWs**	**Skill or Behavior**	**%MWs**
Provide information about postpartum needs including contraception, care of newborn infants, and the importance of exclusive breast feeding for infant health	78%	Provide a safe warm environment for initiating breastfeeding and attachment (bonding) in the first hour of life	76%	Promote early and exclusive breastfeeding while respecting a woman’s choice	84%
Perform a complete physical examination	77%	Provide skin to skin contact and warm environment	73%	Provide and protect privacy and confidentiality for discussions about FP	84%
Respect women’s decisions about participating in treatments and programs	76%	Promote care by mother, frequent feeding and close observation	73%	Provide information about infant needs, frequency and duration of feedings, and weight gain	82%
Counsel women about and offer referral to appropriate persons or agencies for assistance and treatment	75%	Involve partner/ support persons in providing newborn care	73%	Provide support and information about breastfeeding for at least six months	82%
Provide counseling about nutritional supplements such as iron and folic acid, dietary intake, exercise, updating immunizations as needed, modifying risk behaviours, and prevention of sexually transmitted infections, family planning, and methods of contraception.	75%	Ensure clean environment, presence of clean necessary supplies and source of warmth	72%	Provide information to parents about a safe environment for infant, frequent feeding, care of umbilical cord, voiding stooling, and close physical contact	80%
**India**					
**Category 2 (Pre-pregnancy and antenatal care)**		**Category 3 (Care during labor and birth)**		**Category 4 (Ongoing care for women and newborns)**	
**Skill or Behavior**	**%MWs**	**Skill or Behavior**	**%MWs**	**Skill or Behavior**	**%MWs**
Provide information about postpartum needs including contraception, care of newborn infants, and the importance of exclusive breast feeding for infant health	60%	Involve partner/support persons in providing newborn care	68%	Promote early and exclusive breastfeeding while respecting a woman’s choice	74%
Suggest measures to cope with common discomforts of pregnancy	56%	Promote care by mother, frequent feeding, and close observation	64%	Provide information to parents about a safe environment for infant, feeding, care of umbilical cord, voiding and stooling, and close physical contact	72%
Perform a complete physical examination	56%	Provide skin to skin contact and warm environment	60%	Provide information about infant needs, frequency and duration of feedings, and weight gain	70%
Assess nutritional status, current immunization status, health behaviours such as use of substances, existing medical conditions, and exposure to known teratogens	55%	Provide a safe warm environment for initiating breastfeeding and attachment in first hour of life	58%	Identify and manage breastfeeding problems	69%
Discuss (birthplace) options, preferences and contingency plans with woman and support persons and respect their decision	52%	Conduct a complete physical examination of newborn in presence of mother	57%	Provide information to women breastfeeding multiple newborns	67%

**Box 4. The five skills and behaviors least frequently reported for ICM competencies in categories 2–4, by country**

**Table:** 

**Argentina**					
**Category 2 (Pre-pregnancy and ANC)**		**Category 3 (Care during labor and birth)**		**Category 4 (Ongoing care for women and newborns)**	
**Skill or Behavior**	**%MWs**	**Skill or Behavior**	**%MWs**	**Skill or Behavior**	**%MWs**
Mobilizing blood donors	13%	Instituting actions to establish and support breathing and oxygenation	8%	Provide first line measures to treat or stabilize identified conditions in newborn	5%
Providing post-abortion care	20%	Instituting newborn prophylaxis e.g., ophthalmic infection, and hemorrhagic disease, according to policies and guidelines	23%	Examine infant at frequent intervals to monitor growth and developmental behavior	7%
Collecting biological samples for laboratory tests	29%	Undertaking appropriate maneuvers and using maternal position to facilitate vertex, face, or breech birth	24%	Assess newborn infant during postnatal period to detect signs and symptoms of complications	7%
Collaborating in case of complications	29%	Using standardized method to assess newborn conditions in the first few minutes of life	25%	Distinguish normal variation in newborn appearance and behavior from those indicating pathologic conditions	8%
Identifying and assisting people to access and use SRH services	31%	Conducting a complete physical examination of newborn in presence of mother/family	26%	Provide counselling and follow-up care for women and family members who experience stillbirth, neonatal death, serious infant illness, and congenital conditions	8%
**Ghana**					
**Category 2 (Pre-pregnancy and antenatal care)**		**Category 3 (Care during labor and birth)**		**Category 4 (Ongoing care for women and newborns)**	
**Skill or Behavior**	**%MWs**	**Skill or Behavior**	**%MWs**	**Skill or Behavior**	**%MWs**
Carry out screening procedures for STIs and other infections HIV, cervical cancer	46%	Expedite birth in presence of fetal distress	56%	Monitor blood loss and other body functions	62%
Assess fetal size, amniotic fluid volume, fetal position, activity, & heart rate from examination of maternal abdomen	54%	Undertake appropriate manoeuvers and use maternal position to facilitate vertex, face, or breech birth	52%	Provide pain control strategies if needed for uterine contractions, and perineal trauma	64%
Confirm expulsion of products of conception	55%	Prevent unnecessary routine interventions: amniotomy, electronic fetal monitoring, directed closed glottis pushing, episiotomy	56%	Distinguish postnatal depression from transient anxiety about caring for baby, assess availability of help and support at home, and provide emotional support	65%
Collect biologic samples for laboratory tests	58%	Augment uterine contractility judiciously using non-pharmacological or pharmacological agents to prevent non-progressive labor	60%	Provide first line measures to treat or stabilize identified conditions in newborn	65%
Mobilize blood donors if necessary	59%	Institute newborn prophylaxis e.g., ophthalmic infection, and other diseases, according to policies and guidelines	60%	Provide first line measures to treat or stabilize identified conditions in women	66%
**India**					
**Category 2 (Pre-pregnancy and ANC)**		**Category 3 (Care during labor and birth)**		**Category 4 (Ongoing care for women and newborns)**	
**Skill or Behavior**	**%MWs**	**Skill or Behavior**	**%MWs**	**Skill or Behavior**	**%MWs**
Mobilize blood donors if necessary	24%	Provide care for a woman in the birth setting of her choice	25%	Assess woman during postnatal period to detect signs and symptoms of complications	32%
Confirm expulsion of products of conception	26%	Prevent unnecessary routine interventions, e.g., amniotomy, electronic fetal monitoring, directed closed glottis pushing, episiotomy	28%	Distinguish postnatal depression from transient anxiety about caring for baby, assess availability of help and support at home, and provide emotional support	33%
Provide information about conditions that may be detected by screening	27%	Support the woman to give birth in her position of choice	29%	Provide information to woman and family about complications and when to seek help for women	33%
Promote the availability of a full range of birth settings	27%	Augment uterine contractility judiciously using non-pharmacological or pharmacological agents to prevent non-progressive labour	32%	Provide first line measures to treat or stabilize identified conditions in newborn	33%
Participate in/ referring women and support persons to childbirth education program	27%	Order and interpret laboratory tests if needed	32%	Assess mood and feelings about motherhood and demands of infant care	35%

### Midwives’ reported ICM competencies by years of experience and level of facility where employed

Table 5 presents the association between midwives’ self-reported competencies and years of experience as well as facility acuity level. For most skills and behaviors, midwives with greater experience more frequently reported possessing the skill, with some variation between the countries. In Argentina, for the subcategory “*assess fetal well-being”*, the percent of midwives who reported possessing the associated skills was 42% among those with five years’ experience and 68% among those with 10 years’ experience. Similarly, in Ghana, the percent of midwives who reported possessing the associated skills was in the same subcategory was 46% among those with five years’ experience and 72% for those with more than 10 years’ experience. In India, 10% of midwives with 5 years of experience and 24% of midwives with more than 10 years of experience reported they had all the skills for the same subcomponent. In Ghana, respondents with more than 10 years’ experience reported possessing the greatest number of skills and behaviors across all ICM Categories compared to experienced midwives in Argentina or India.

**Table 5:** Percent midwives who reported having all the required skills for specific ICM competencies, by number of years of practice and type of facility where employed.

	Argentina						Ghana					India					
	Years of Experience of Midwives			Level of facility Midwives are working			Years of Experience of Midwives			Level of facility Midwives are working		Years of Experience of Midwives			Level of facility Midwives are working		
	<5 yrs N = 12	5–10 yrs N = 31	>10 yrs N = 40	I N = 7	II N = 16	III N = 63	<5 yrs N = 168	5–10 yrs N = 150	>10 yrs N = 82	I	II	<5 yrs N = 199	5–10 yes N-293	>10 yrs N = 274	I N = 208	II N = 425	III N = 133
**Category 2: Provide Pre-pregnancy and Antenatal Care**																	
2.a Provide pre-pregnancy care	25.0 (3)	16.1 (5)	22.5 (9)	28.6 (2)	25 (4)	17.5 (11)	49.7 (84)	46 (69)	63.4 (52)	53.6 (187)	35.4 (23)	7.0 (14)	10.6 (31)	15.3 (42)	11.5 (24)	11.1 (47)	12.0 (16)
2.b Determine health status of woman	16.6 (2)	3.2 (1)	17.5 (7)	14.3 (1)	12.5 (2)	11.1 (7)	-	-	-	44.1 (154)	32.3 (21)	10.1 (20)	14.7 (43)	21.9 (60)	9.6 (20)	13.9 (59)	33.1 (44)
2.c Assess fetal well-being	41.6 (5)	64.5 (20)	67.5 (27)	71.4 (5)	62.5 (10)	61.9 (39)	45.7 (77)	49.3 (74)	72.0 (59)	49.6 (173)	64.6 (42)	9.6 (19)	13.7 (40)	24.1 (66)	7.7 (16)	18.4 (78)	23.3 (31)
2.d Monitor progression of pregnancy	25.0 (3)	22.5 (7)	50 (20)	28.6 (2)	37.5 (6)	34.9 (22)	55.6 (94)	52.7 (79)	73.2 (60)	56.5 (197)	63.1 (41)	5.0 (10)	12.3 (36)	27.0 (74)	15.9 (33)	14.8 (63)	18.1 (24)
2.e Promote and support health behaviors that improve well being	25.0 (3)	16.1 (5)	30 (12)	28.6 (2)	6.3 (1)	27.0 (17)	63.3 (107)	56 (84)	69.5 (57)	62.8 (219)	53.9 (35)	10.1 (20)	13.0 (38)	16.1 (44)	14.9 (31)	11.3 (48)	17.3 (23)
2.f Provide anticipatory guidance related to pregnancy, birth, breastfeeding, parenthood, and change in the family	50.0 (6)	22.5 (7)	50 (20)	71.4 (5)	31.3 (5)	36.5 (23)	56.8 (96)	55.3 (83)	70.7 (58)	60.2 (210)	49.2 (32)	12.1 (24)	14.0 (41)	15.3 (42)	13.5 (28)	12.5 (53)	19.6 (26)
2.g Detect, stabilise, manage, and refer women with complicated pregnancies	0 (0)	6.4 (2)	15 (6)	14.3 (1)	12.5 (2)	7.9 (5)	44.4 (75)	44 (66)	59.8 (49)	47.3 (165)	44.6 (29)	3.5 (7)	7.9 (23)	12.0 (33)	9.1 (19)	7.1 (30)	10.5 (14)
2.h Assist the woman and her family to plan for an appropriate place of birth	16.6 (2)	25.8 (8)	50 (20)	57.1 (4)	50 (8)	30.2 (19)	58.6 (99)	58.7 (88)	68.3 (56)	59.0 (206)	64.6 (42)	5.0 (10)	14.7 (43)	25.2 (69)	8.2 (17)	16.2 (69)	27.1 (36)
2.i Provide care to women with unintended or mistimed pregnancy	8.3 (1)	6.4 (2)	15 (6)	14.3 (1)	6.3 (1)	11.1 (7)	39.1 (66)	44.7 (67)	59.8 (49)	46.7 (133)	36.9 (24)	4.5 (9)	7.2 (21)	9.9 (27)	2.9 (6)	6.6 (28)	17.3 (23)
**Category 3: Care During Labor & Birth **																	
3.a Promote physiologic labour and birth	25.0 (3)	25.8 (8)	35 (14)	14.3 (1)	18.8 (3)	33.3 (21)	36.7 (62)	35.3 (53)	45.1 (37)	38.1 (133)	35.4 (23)	1.5 (3)	3.8 (11)	8.0 (22)	1.0 (2)	4.2 (18)	12.0 (16)
3.b Manage a safe spontaneous vaginal birth and prevent complications	0 (0)	19.3 (6)	17.5 (7)	0 (0)	6.3 (1)	19.1 (12)	29.6 (50)	36.7 (55)	53.7 (44)	37.5 (131)	35.4 (23)	1.1 (2)	2.4 (7)	9.9 (27)	1.4 (3)	4.7 (20)	9.8 (13)
3.c Provide care of the newborn immediately after birth	0 (0)	6.4 (2)	10 (4)	0 (0)	6.3 (1)	7.9 (5)	46.6 (78)	49.3 (74)	63.4 (52)	48.4 (169)	61.5 (40)	18.1 (36)	30.0 (88)	41.6 (114)	29.8 (62)	33.4 (142)	25.6 (34)
**Category 4: Ongoing Care of Women and Newborns**																	
4.a Provide postnatal care for healthy woman	25.0 (3)	29.0 (9)	47.5 (19)	71.4 (5)	18.8 (3)	38.1 (24)	52.1 (88)	48.0 (72)	65.9 (54)	51.9 (181)	58.5 (38)	10.1 (20)	16.7 (49)	29.9 (82)	15.9 (33)	20.7 (88)	22.6 (30)
4.b Provide care to healthy newborn infant	0 (0)	3.2 (1)	2.5 (1)	0 (0)	6.3 (1)	1.6 (1)	61.0 (103)	62 (93)	72.0 (59)	64.2 (224)	56.9 (37)	11.1 (22)	24.9 (73)	38.7 (106)	17.8 (37)	29.4 (125)	29.3 (39)
4.c Promote and support breastfeeding	16.6 (2)	16.1 (5)	30 (12)	28.6 (2)	18.8 (3)	22.2 (14)	69.8 (118)	65.3 (98)	76.8 (63)	67.6 (236)	73.9 (48)	21.1 (42)	34.5 (101)	51.5 (141)	22.6 (47)	41.8 (178)	44.4 (59)
4.d Detect, treat, and stabilize postnatal complications in woman and refer as necessary	8.3 (1)	9.6 (3)	7.5 (3)	0 (0)	6.3 (1)	9.5 (6)	51.5 (87)	54.0 (81)	72.0 (59)	56.7 (198)	52.3 (34)	6.0 (12)	15.0 (44)	29.2 (80)	25.5 (53)	15.5 (66)	12.8 (17)
4.e Detect, stabilize, and manage health problems in newborn infant and refer if necessary	0 (0)	3.2 (1)	2.5 (1)	0 (0)	0 (0)	3.2 (2)	49.1 (83)	58.0 (87)	63.4 (52)	54.7 (191)	55.4 (36)	11.2 (22)	21.5 (63)	35.0 (96)	33.2 (69)	21.7 (92)	15.0 (20)
4.f Provide family planning services	25.0 (3)	32.2 (10)	47.5 (19)	57.1 (4)	6.3 (1)	44.4 (28)	62.7 (106)	58.0 (87)	69.5 (57)	63.3 (221)	56.9 (37)	28.1 (56)	42.3 (124)	50.0 (137)	49.5 (103)	40.7 (173)	30.8 (41)

* The cells have less than 5 or less sample.

Table 5 also displays differences in reported skills by the facility level where midwives worked. More respondents employed in primary care facilities in Argentina reported possessing the skills for the Category 2 subcategory, *‘provide pre-pregnancy care’* (29%) than respondents in secondary or tertiary facilities (25% and 18%, respectively). However, for Category 3, none of the midwives working at primary care level in Argentina reported having skills included within the subcategory, ‘*manage a safe spontaneous vaginal birth and prevent complications*’ compared to 19% in secondary level facility and 30% in a tertiary facility. For Category 4 in Argentina, the percentage of midwives reporting that they have the skills within the subcategory necessary to *‘provide postnatal care for the healthy woman’* at primary, secondary and tertiary level were 71%, 19% and 38% respectively.

In Ghana, there are two levels of facility where midwives work. More midwives from secondary facilities (60%) than from primary facilities (50%) reported that they had the skills within the subcategory ‘*assess fetal wellbeing’* from ICM Category 2. Conversely, 60% of midwives from primary facilities and 49% from secondary facilities in Ghana reported that they had all the skills in the subcategory necessary to *‘provide anticipatory guidance related to pregnancy*, *birth*, *breastfeeding*, *parenthood*, *and change in the family’*. For Category 3, a similar percentage of midwives at primary (38%) and secondary (35%) facilities in Ghana reported possessing the skills to *‘manage a safe spontaneous vaginal birth’*. For ICM Category 4, there was minimal variation in the percentage of midwives’ reports between facility levels.

For Category 2 in India, the percentages of respondents who reported possessing skills matching all the ICM sub-categories were low overall but did not vary greatly across the three facility levels. For example, the skills under the subcategory necessary to *‘monitor the progression of labor’* were reported by 16%, 15%, 18% of midwives across primary, secondary, and tertiary levels respectively. For Category 3, the percentage of midwives who reported that they had all necessary skills under the subcategory to *‘provide care to healthy newborn infant’* was 30% at primary level, 33% at secondary level and 26% at tertiary level. For Category 4 under the subcategory ‘*provide family planning services’*, 49% of midwives at the primary facility level reported they could, compared to 41% of midwives at the secondary level and 31% of midwives at the tertiary level.

### Where skills related to ICM competencies were gained

Table 6 describes the distribution of where midwives gained their skills aligned to the ICM Competencies (during pre-service training, in-service training, or OJT), with variation observed between countries and ICM categories. Multiple response categories were permitted. In Argentina, more midwives reported gaining their skills after pre-service education, either through continuing education or OJT. Conversely, midwives in Ghana and India reported gaining most skills related to ICM competencies during their pre-service education as well as OJT.

**Table 6:** Percent of midwives who reported ICM competencies by the source where skills were gained*.

	Argentina (n = 86)			Ghana (n = 414)			India (n = 766)		
Type of services provided	Pre-Service education/ school	In-service/ continuing education	On the job experience	Pre-Service education/ school	In-service/ continuing education	On the job experience	Pre-Service education/ school	In-service/ continuing education	On the job experience
**Category 2: Provide Pre-pregnancy and Antenatal Care **									
2.a Provide pre-pregnancy care	57.6	83.5	70.5	82.7	77.2	84.9	95.2	72.3	93.2
2.b Determine health status of woman	57.6	83.5	74.1	78.2	74.5	84.4	100.0	70.8	95.3
2.c Assess fetal well-being	62.3	81.1	80.0	83.1	70.2	87.6	92.3	70.2	95.4
2.d Monitor the progression of pregnancy	68.2	85.8	77.6	82.1	70.2	86.4	100.0	71.7	93.9
2.e Promote and support health behaviours that improve well being	58.3	76.1	77.3	79.8	67.4	84.9	87.5	70.5	94.9
2.f Provide anticipatory guidance related to pregnancy, birth, breastfeeding, parenthood, and change in the family	61.1	82.3	76.4	82.0	68	85.8	61.6	68.7	94.8
2.g Detect, stabilise, manage, and refer women with complicated pregnancies	54.1	84.7	85.8	79.2	70.2	87.3	65.0	70.0	95.8
2.h Assist the woman and her family to plan for an appropriate place of birth	37.3	65.0	75.9	81.6	66.2	84.3	94.7	69.2	95.3
2.i Provide care to women with unintended or mistimed pregnancy	37.6	90.5	76.4	81.3	68.3	84.4	62.3	73.2	93.4
**Category 3: Care During Labor & Birth**									
3.a Promote physiologic labour / birth	63.1	78.5	84.5	80.4	66.5	85.6	90.3	70.2	94.9
3.b Manage a safe spontaneous vaginal birth and prevent complications	63.1	82.1	92.8	81.0	68.3	85.8	87.5	70.4	95.4
3.c Provide care of the newborn immediately after birth	55.9	72.6	84.5	83.6	70.8	85.2	92.0	70.4	94.9
**Category 4: Ongoing Care of Women and Newborns**									
4.a Provide postnatal care for healthy woman	66.6	84.5	83.3	84.2	72	84.9	91.3	70.4	94.8
4.b Provide care to healthy newborn infant	56.1	71.9	75.6	83.3	67.5	86.2	88.6	70.8	95.8
4.c Promote and support breastfeeding	58.3	83.3	82.1	85.4	72.3	87.6	100.0	71.4	93.2
4.d Detect, treat, stabilise postnatal complications in woman and refer as necessary	50.0	78.5	82.1	81.5	66.8	85.5	66.1	70.0	95.0
4.e Detect, stabilise, and manage health problems in newborn infant and refer if necessary	48.1	62.9	67.9	78.4	65.3	82.7	64.5	70.4	94.8
4.f Provide family planning services	60.4	83.9	81.4	83.6	71.6	86	100.0	70.2	96.3

* Multiple response question

## Discussion

This study provides data critical for validating measures to assess the adequacy of the midwifery workforce in three diverse countries. This is, to our knowledge, the first study to examine the adoption of global standards for midwifery scope of practice and competency within national policies and guidelines for the regulation of midwives’ job tasks, skills, and behaviors. Previously published studies [6, 23] have not comprehensively assessed standards at national level in comparison to both ILO tasks and ICM competencies. Our study evaluates the reliability of the numerator in measures of midwifery density and the consistency with which midwives’ scope of practice and competencies to provide MNH care reflect global benchmarks at the level of both policy and practice. In Argentina, Ghana, and India, we compared the regulatory scopes of practice against international reference standards from the ILO ISCO-8 and Categories 2, 3, and 4 of the ICM Essential Competencies for Basic Midwifery Practice. We also assessed midwives’ self-reported skills and behaviors in relation to the ICM competencies, and where those skills were gained.

The overall findings of this study suggest a considerable variation in national scopes of practice for midwifery professionals compared to the ILO ISCO-8 across the three study countries. The broadest scope of practice is observed in Ghana and the most restricted is in Argentina. The national law that currently regulates the professional practice of midwives in Argentina dates from 1967 when the role of midwives was limited to merely collaborating with physicians, and thus it authorizes them to provide only partial, fragmented care to women before, during, and after pregnancy and childbirth. The Argentinian federal scope of practice for midwifery professionals covers only two out of eight ILO tasks (Table 1). The national law still does not allow midwives to perform the competencies learned through the current national curriculum for undergraduate university-based training programs with full autonomy, thus limiting their scope of work. However, as Argentina is a federal country, several provinces—who have the power to generate their own laws—have updated their scopes of practice. A specialized health professional cadre in Argentina provides newborn care; thus, the policy governing service delivery does not yet reflect the broader global standards that would allow midwives to exercise their skills across the pregnancy-postnatal continuum.

In Ghana there was consistent agreement between the National Reproductive Health Policy and Standards and the ILO task list. Ghana’s Free Maternal Health Policy has been in existence since 2008 and there has been notable progress in increasing the number of midwives and other health staff and reducing maternal and newborn mortality [24]. In 2021, Ghana launched a comprehensive Reproductive, Maternal, Newborn, Child and Adolescent Health Strategic Plan with a focus on increasing access to quality services [25]. This strategic plan provides an overarching framework for several strategies including the National Reproductive Health Service Policy and Standards—demonstrating Ghana’s commitment to expansion of a relevant MNH workforce.

The skills reported by India’s midwifery workforce overall matched across the ILO ISCO-8 Tasks with some inconsistencies. To address the gaps in providing quality MNH care in India, there has been a move–although still nascent—towards developing a midwifery cadre who can work independently, providing care of women and newborns at midwife-led birth centers, with support from doctors when complications are identified. The Government of India has committed to adding an additional 85,000 midwives [26], including drafting new policy documents at national level regarding midwifery practice; however, these are yet to be legislated and adopted formally. The first tranche of trained midwives is expected to qualify in 2024.

In a multi-country (n = 73) review of midwifery education, regulation, and association, less than half of the countries had legislation that recognized midwifery as an independent profession [27]. The inconsistent application of definitions of midwifery and varying midwifery scopes of practice have resulted in a mixture of professional and associate professional cadres who do not have the scope or skills to provide all the midwifery behaviors reflecting the essential competencies for basic midwifery practice that are required for comprehensive MNH care [3].

Findings from the three countries in our study show considerable variation of reported skills and behaviors across the three categories of ICM competencies studied. In all three study countries, midwifery scopes of practice mostly aligned with the skills and behaviors for ICM competencies that fall within Category 3 (Care During Labor and Birth). We know, however, that midwives offer more than just care during labor and birth [28]. This finding may be attributed to the fact that in many policy frameworks, labor and delivery care still represents the bulk of the midwifery scope of practice, despite international calls for midwives’ roles to be expanded across the full spectrum of evidence based reproductive and MNH responsibilities [3].

In a push to reduce maternal and newborn morbidity and mortality to meet both the United Nations Millennium Development Goals and Sustainable Development Goals there has been much focus over the last two decades on updating health provider skills around “the day of birth”. Numerous development and bilateral donors have prioritized investments supporting in-service training on Emergency Obstetric and Newborn Care in multiple countries, without necessarily aligning such efforts with each country’s policy and health worker regulatory processes [29]. Adopting global recommendations, changing policy, and subsequently revising clinical guidelines and health information systems to capture data at the country level is a slow process. In addition to narrowly targeting focus on emergency functions, many countries designated new cadres of health workers to serve as skilled birth attendants (SBAs); while well-intentioned, both strategies can mask limitations in providing quality of care due to the lack of requisite comprehensive midwifery skills [30, 31]. Moreover, the SBA indicator used in Demographic and Health Surveys was not validated until 2015 [21]—potentially misrepresenting the number of women whose births were attended by skilled personnel.

A study in Middle East and North African countries indicated that midwives from different nations have non-standardized levels of profisciency, scopes of practice, and education [6]. However, with a growing skilled midwifery workforce across the world, Renfrew and colleagues argue that there can be a system-level shift from MNH focused on identification and treatment of pathology for the minority to skilled care for all [3]. In our study, this shift toward a broader scope of midwifery practice was evident in Ghana, where more than half of the respondents reported having all the necessary skills for the three ICM skill categories. However, at the same time in Ghana, there was a small but significant number of survey participants who were screened in as eligible based on confirmation that they perform the ILO classification tasks in their current job but were from cadres that are not regulated to provide midwifery care. These cadres include registered and enrolled nurses, nutrition nurses, community health nurses and officers, and field technicians among others [32, 33]. This underscores the midwifery shortage and the need for valid measures of availability of skilled midwifery professionals.

In India—perhaps unsurprisingly, given the lack of an independent cadre of professional midwives—fewer respondents possess all the basic skills for intrapartum care competencies. There are gaps in reported skills in critical areas for detecting, treating, and stabilizing pregnancy related complications. This finding supports a systematic review in India which suggests that lack of competency of midwives is a key barrier to the provision of high-quality midwifery services in India [34]. The overall percentages are quite low on several of the essential competencies, contrasting reports of higher percentages for specific ICM skills, which demonstrate the potential and the capacity for improving midwifery skills and behaviors if opportunities were available.

Knowledge gained from pre-service theory, coupled with OJT experience is essential to improve the competencies of the midwives at their place of work. There were differences between countries in midwives’ reports of where they acquired the necessary skills and behaviors to align with the ICM competencies. Midwives from Argentina reported acquiring more skills through in-service education and OJT experience–interestingly—even though most respondents from Argentina are university graduates. In Ghana and India midwives reported gaining more skills through pre-service training and OJT experience. OJT experience was high across all three countries for specific subcategories of competencies in Category 3—such as ‘*manage a safe spontaneous vaginal birth and preventing complications’*, where 93% in Argentina, 86% in Ghana and 95% in India reported gaining the skills via this modality. Elsewhere in Ghana, a study found inadequate in-service training and midwives felt they missed opportunities for continuing education as well as having limited knowledge of relevant health policies [24, 32]. This might explain why midwives reported learning skills in pre-service training and OJT practice in Ghana rather than through in-service training.

A multi-country study, including Argentina, India, and several African countries, found that midwives in Argentina had more years of labor room experience on average than midwives in other countries, giving them greater confidence in their clinical practice [23], which is consistent with the results of this study. On-the-job learning, which can focus on specific gaps in skills and behaviors, may be a more useful, cost-effective approach to improve midwives’ performance in providing quality care [35].

Global standards in the form of the ICM Competencies lay out the core skills and behaviors that constitute competency to perform the midwifery scope of practice and can be used as benchmarks to indicate the adequacy of the midwifery workforce, considering effective coverage and expected quality of care. For this study we used the skills and behaviors part of the ICM Competencies, Categories 2, 3, and 4, as the skills and behaviors are the most objective, observable aspect of these standards. The skills and behaviors listed in some of the ICM core competencies are however very detailed, complex, and diverse. There are 18 subcategories and over 110 components within the three ICM categories assessed. Each component is a complex composite of numerous skills and tasks that are not assigned value or a scoring system and do not obviously contribute to one discrete domain of competency. As the predominant set of globally recognized standards or benchmarks, these competencies are not well constructed from a measurement standpoint. Not surprisingly, midwives in this study more frequently reported possessing skills to match the more easily understood, discrete, and measurable sub-components–such as *‘assess fetal movements’*, *‘provide skin-to-skin contact’* and *‘promote early and exclusive breastfeeding’* over those skills and behaviors that are more complex or comprise numerous, unrelated elements, such as: *‘provide counselling about nutritional supplements such as iron and folic acid*, *dietary intake*, *exercise*, *updating immunizations as needed*, *modifying risk behaviours*, *and prevention of sexually transmitted infections*, *family planning*, *and methods of contraception’*

Our study findings from three countries demonstrate gaps in both midwifery policy and practice. Nevertheless, there is a growing evidence base to demonstrate that when midwives have opportunities to be trained to global standards, licensed and regulated, and working within functional systems and enabling environments, they can provide most essential RMNH services [1, 5, 14] and components of Quality Maternal and Newborn Care [2, 3]. When these conditions are met, care provided by midwives and midwife-led care demonstrate comparable or better outcomes than other models of care. This includes studies demonstrating women were more likely to have a normal vaginal birth and less likely to experience pain, and more likely to be attended at birth by a known midwife (among others) [5, 36]. It is estimated that over 60% of all maternal and neonatal deaths and stillbirths could be prevented if there was universal coverage of known midwifery interventions [37]. This rises to 83% of maternal, perinatal, and neonatal deaths that could be prevented when midwifery care combines MNH interventions and family planning [37]. Our study results show that implementing the full scope of midwifery practice, with the potential gains associated, is a goal that is not being met in our research settings.

From a policy perspective, country guidelines can be modified to fully authorize midwives to perform all the recommended tasks outlined by ILO and ICM that are essential for improving MNH outcomes and be supported to do so. From a country program perspective, curriculum development can be tailored to build midwives’ skills and adopt competency-based learning across the whole maternity care continuum. Pre-service and in-service midwifery training programs, and other MNH in-service updates have been developed in many countries, however there is not always a systematic process within human resource departments to ensure all midwives have opportunities to access in-service training or OJT mentoring to improve their skills and behaviors especially at primary and secondary facilities.

It appears that where national documents are more closely aligned with ICM competencies such as in Ghana, midwives report more skills and behaviors to match the ICM standards for the three categories. In Argentina, midwives reported higher percentages matching specific antenatal and intrapartum component, suggesting potential to gain competency in other areas if regulation allowed. Overall, in India, midwives reported lower percentages of skills and behaviors matching ICM standards–however many reported higher percentages for specific skills–again indicating that their full potential has yet to be met.

While the study offers important insights into the midwifery scope of practice in each of the study countries, the findings may be interpreted with caution. First, while an extensive search of national documents relevant to midwifery regulations and competencies in each country was undertaken, there is a possibility that some national policy documents were missed. Second, the self-reported skills and behaviors by the midwives in the survey may have been under- or over-reported. Third, the ICM competencies, the “yardstick” used to measure competency may be subject to measurement error, given the complexity of considering all composite skills and behaviors in each sub-category (i.e., *“I have all the competencies”*). Moreover end-aversion bias (i.e., participants avoid extreme choices on a scale and select a neutral middle of scale option) may have influenced participants responses differently, including according to their culture. Finally, the method for data collection across the three study countries was not uniform, due to the global COVID-19 pandemic, thus response bias may have affected the study results.

Nevertheless, this is, to our knowledge, the first study to examine the adoption of global standards (ILO tasks and ICM competencies) within national policy and guidelines regulating the practice of midwifery in three countries. Previous studies did not comprehensively compare standards to both ILO and ICM standards [6, 23]. We also examined midwives’ reported skills and behaviors in Categories 2–4 of the ICM competencies and highlighted the gaps in skills and behaviors in three different contexts. Our findings demonstrate challenges associated with both meeting and measuring the standards for midwifery scope of practice and competency that should provide a foundation for estimating effective coverage of reproductive and MNH services by the midwifery workforce (“i.e., the proportion of the population who have need of an intervention and receive that intervention with sufficient quality to be effective, and benefit from it”) [3, 14, 38]. Coverage of midwifery care goes beyond the raw number of designated midwives in the workforce and includes demonstrable competency within a supportive policy and health system environment that ensures access to appropriate pre-service education, in-service training and OJT skills updates [3]. Only if midwifery professionals in the count reliably demonstrate the ability to exercise the same scope and skills with comparable competency can we assume that that the number of midwives in the numerator can be taken as a valid indicator of an adequate midwifery workforce, considering effective coverage and the expected quality of care.

## Supporting information

S1 Table All skills assessment of ICM behaviors in category 2- provide pre-pregnancy and ANC. (DOCX)

S2 Table All skills assessment of ICM behaviors in category 3: Care during labor & birth. (DOCX)

S3 Table All skills assessment of ICM behaviors in category 4: Ongoing care of women and newborns. (DOCX)

S1 File Inclusivity in global research. ss
(DOCX)

## Abbreviations

ANC: Antenatal Care
ANM: Auxiliary Nurse Midwife
COVID-19: Coronavirus Disease of 2019
EmONC: Emergency Obstetric and Newborn Care
EPMM: Ending Preventable Maternal Mortality
FP: Family Planning
HIV: Human Immunodeficiency Virus
ICM: International Confederation of Midwives
ILO: International Labour Organization
ISCO-8: International Standard Classification of Occupations 2008
LHV: Lady Health Visitor
MNCAH: Maternal Newborn Child and Adolescent Health
MNH: Maternal and Newborn Health
MW: Midwives
OJT: On (the) Job Training
PNC: Postnatal Care
QMNC: Quality Maternal and Newborn Care
SN: Staff Nurse
UNFPA: United Nations Population Fund
WHO: World Health Organization
