# Supplementary material for: Validating midwifery professionals’ scope of practice and competency: A multi-country study comparing national data to international standards
Source: PLoS One. 2023 May 25;18(5):e0286310. doi: 10.1371/journal.pone.0286310 (PMC10212099; doi:10.1371/journal.pone.0286310)
Supplement: S1 Table — (DOCX) [file pone.0286310.s001.docx]

**S1 Table**

**Table 3A: All Skill Assessment of ICM Behaviours for Midwives in Category 2- Provide Pre-pregnancy and Antenatal Care**

|  | Argentina | Ghana | India |
| --- | --- | --- | --- |
| Total number of midwives | 86 | 414 | 766 |
|  | % | % | % |
| 2.a. Provide Pre-pregnancy Care | 19.8 | 34.5 | 11.3 |
| - Identify and assist in reducing barriers related to accessing and using sexual and reproductive health services | 31.4 | 63.5 | 28.9 |
| - Assess nutritional status, current immunization status, health behaviours such as use of substances, existing medical conditions, and exposure to known teratogens | 34.9 | 60.6 | 34.9 |
| - Carry out screening procedures for sexually transmitted and other infections, HIV, cervical cancer | 54.7 | 45.7 | 46.5 |
| - Provide counselling about nutritional supplements such as iron and folic acid, dietary intake, exercise, updating immunizations as needed, modifying risk behaviours, and prevention of sexually transmitted infections, family planning, and methods of contraception | 69.8 | 74.6 | 46.0 |
| 2.b. Determine Health Status of Women | 11.6 | 42.2 | 16.1 |
| - Confirm pregnancy and estimate gestational age from history, physical exam, laboratory test and/or ultrasound | 88.4 | 68.8 | 36.6 |
| - Obtain comprehensive health history | 61.6 | 72.0 | 48.0 |
| - Perform a complete physical examination | 33.7 | 76.6 | 56.4 |
| - Biologic samples for laboratory tests (e.g., venepuncture, finger puncture, urine samples, and vaginal swabs) | 29.1 | 57.7 | 27.6 |
| - Provide information about conditions that may be detected by screening | 53.5 | 63.5 | 26.8 |
| - Assess status of immunizations, and update as indicated | 66.3 | 72.9 | 55.4 |
| - Discuss findings and potential implications with woman and mutually determine plan of care | 58.1 | 69.1 | 36.3 |
| 2.c. Assess Fetal Well-being | 62.8 | 51.9 | 16.3 |
| - Assess fetal size, amniotic fluid volume, fetal position, activity, and heart rate from examination of maternal abdomen | 72.1 | 53.9 | 27.6 |
| - Determine whether there are indications for additional assessment/examination and refer accordingly | 74.4 | 66.4 | 31.5 |
| - Assess fetal movements and ask woman about fetal activity | 86.1 | 73.4 | 36.4 |
| 2.d. Monitor the Progression of Pregnancy | 34.9 | 57.5 | 15.7 |
| - Conduct assessments throughout pregnancy of woman’s physical and psychological well-being, family relationships, and health education needs | 41.9 | 70.8 | 50.0 |
| - Provide information regarding normal pregnancy to woman, her partner, family members, or other support persons | 68.6 | 72.2 | 48.2 |
| - Suggest measures to cope with common discomforts of pregnancy | 61.6 | 72.5 | 56.5 |
| - Provide information (including written and/or pictorial) about danger signs, (e.g., vaginal bleeding, signs of preterm labour, prelabour, rupture of membranes) emergency preparedness, and when and where to seek help | 87.2 | 69.8 | 43.1 |
| - Review findings and revise plan of care with woman as pregnancy progresses | 62.8 | 65.5 | 34.6 |
| 2.e Promote and support health behaviours that improve well being | 23.3 | 61.4 | 13.3 |
| - Provide emotional support to women to encourage change in health behaviour | 41.9 | 74.4 | 37.9 |
| - Provide information to woman and family about impact on mother and fetus of risk conditions | 53.5 | 73.2 | 42.3 |
| - Counsel women about and offer referral to appropriate persons or agencies for assistance and treatment | 59.3 | 75.1 | 43.2 |
| - Respect women’s decisions about participating in treatments and programs | 53.4 | 75.6 | 37.3 |
| - Make recommendations and identify resources for smoking reduction/cessation in pregnancy | 31.4 | 66.7 | 30.9 |
| 2.f Provide anticipatory guidance related to pregnancy, birth, breastfeeding, parenthood, and change in the family | 38.4 | 58.5 | 14.0 |
| - Participate in--and refer women and support persons to--childbirth education programs | 76.7 | 69.6 | 27.2 |
| - Convey information accurately and clearly and respond to needs of individuals | 70.9 | 72.0 | 34.1 |
| - Prepare the woman, partner, and family to recognize labour onset, when to seek care, and progress of labour | 83.7 | 73.2 | 38.0 |
| - Provide information about postpartum needs including contraception, care of new-born infants, and the importance of exclusive breastfeeding for infant health | 79.1 | 77.5 | 60.4 |
| - Identify needs or problems requiring further expertise or referral such as excessive fear, and dysfunctional relationships | 46.5 | 65.7 | 33.6 |
| 2.g Detect, stabilise, manage, and refer women with complicated pregnancies | 9.3 | 46.9 | 8.2 |
| - Stabilise in emergencies and refer for treatment as necessary | 36.1 | 63.5 | 28.5 |
| - Collaborate in care of complications | 29.1 | 62.3 | 40.3 |
| - Implement critical care activities to support vital body functions (e.g., intravenous (IV) fluids, magnesium sulphate, antihemorrhagics) | 45.4 | 61.4 | 29.9 |
| - Mobilize blood donors if necessary | 12.8 | 59.4 | 24.5 |
| - Transfer to higher level facility if needed | 66.3 | 70.3 | 32.4 |
| 2.h Assist the woman and her family to plan for an appropriate place of birth | 36.1 | 46.9 | 15.9 |
| - Discuss options, preferences and contingency plans with woman and support persons and respect their decision | 52.3 | 69.8 | 52.0 |
| - Provide information about preparing birth site if in community, e.g., travel and admission to facility | 44.2 | 69.1 | 46.4 |
| - Promote the availability of a full range of birth settings | 45.4 | 64.7 | 27.0 |
| 2.i Provide care to women with unintended or mistimed pregnancy | 10.5 | 45.2 | 7.4 |
| - Provide information about abortion | 39.5 | 63.3 | 35.9 |
| - Refer to a provider of abortion services upon request | 61.6 | 66.4 | 27.9 |
| - Provide post-abortion care including psychological response to abortion | 19.8 | 61.8 | 31.5 |
| - Confirm expulsion of products of conception from history, ultrasound, or levels of HCG | 33.7 | 54.6 | 26.2 |
| - Review options for contraception and initiate immediate use of method | 67.4 | 68.6 | 28.7 |
