# Supplementary material for: Validating midwifery professionals’ scope of practice and competency: A multi-country study comparing national data to international standards
Source: PLoS One. 2023 May 25;18(5):e0286310. doi: 10.1371/journal.pone.0286310 (PMC10212099; doi:10.1371/journal.pone.0286310)
Supplement: S2 Table — (DOCX) [file pone.0286310.s002.docx]

**S2_Table**

**Table 3B: All Skill Assessment of ICM Behaviours for Midwives in Category 3: Care During Labor & Birth**

|  | **Argentina** | **Ghana** | **India** |
| --- | --- | --- | --- |
| Total number of midwives | **86** | **414** | **766** |
|  | % | % | % |
| **3.a. Promote physiologic labour and birth** |  |  |  |
| - Provide care for a woman in the birth setting of her choice, following policies and protocols | 83.7 | 37.7 | 4.7 |
| - Obtain relevant obstetric and medical history? | 80.2 | 66.7 | 24.9 |
| - Perform and interpret focused physical examination of woman & fetus? | 64.0 | 66.4 | 40.5 |
| - Order and interpret laboratory tests if needed? | 67.4 | 66.9 | 41.3 |
| - Assess woman’s physical and behavioural responses to labour? | 45.4 | 62.6 | 32.1 |
| - Provide information, support, and encouragement to woman and support persons throughout labour and birth | 69.8 | 67.9 | 40.6 |
| - Provide respectful one-to-one care | 72.1 | 69.1 | 40.5 |
| - Encourage freedom of movement and upright positions | 68.6 | 71.7 | 49.7 |
| - Provide nourishment and fluids | 60.5 | 65.9 | 45.8 |
| - Offer and support woman to use strategies for coping with labor pain, e.g., controlled breathing, water immersion, relaxation, massage, and pharmacologic modalities when needed | 61.6 | 66.9 | 54.4 |
| - Assess regularly parameters of maternal-fetal status, e.g., vital signs, contractions, cervical changes, and fetal descent | 88.4 | 63.8 | 42.8 |
| - Use labour progress graphic display to record findings and assist in detecting complications, e.g., labour delay, fetal compromise, maternal exhaustion, hypertension, infection | 77.9 | 64.5 | 49.2 |
| - Augment uterine contractility judiciously using non-pharmacological or pharmacological agents to prevent non-progressive labour | 74.4 | 59.7 | 38.5 |
| - Prevent unnecessary routine interventions, e.g., amniotomy, electronic fetal monitoring, directed closed glottis pushing, episiotomy |  | 55.6 | 31.5 |
| **3.b Manage a safe spontaneous vaginal birth and prevent complications** |  |  |  |
| - Support the woman to give birth in her position of choice | 68.6 | 63.5 | 28.9 |
| - Ensure clean environment, presence of clean necessary supplies and source of warmth | 60.5 | 72.2 | 33.2 |
| - Coach woman about pushing to control expulsion of presenting part, avoid routine episiotomy | 75.6 | 67.9 | 33.3 |
| - Undertake appropriate manoeuvres and use maternal position to facilitate vertex, face, or breech birth | 24.4 | 52.4 | 35.8 |
| - Expedite birth in presence of fetal distress | 52.3 | 55.6 | 35.6 |
| - Delay cord clamping | 84.9 | 60.1 | 45.2 |
| - Manage nuchal cord | 72.1 | 58.0 | 37.6 |
| - Assess immediate condition of newborn | 32.6 | 65.9 | 45.2 |
| - Provide skin to skin contact and warm environment | 80.2 | 73.4 | 59.7 |
| - Deliver placenta and membranes and inspect for completeness | 88.4 | 65.5 | 50.9 |
| - Assess uterine tone, maintain firm contraction, and estimate and record maternal blood loss; manage excessive blood loss including administration of uterotonics | 76.7 | 59.7 | 40.9 |
| - Inspect vaginal and perineal areas for trauma, and repair as needed, following policies and protocols | 75.6 | 60.6 | 33.8 |
| - Refer for continuing treatment of any complications as needed | 79.1 | 66.4 | 37.6 |
|  |  |  |  |
| **3.c Provide care of the newborn immediately after birth** |  |  |  |
| - Use standardized method to assess newborn condition in the first minutes of life (Apgar or other); refer if needed | 25.6 | 65.7 | 53.3 |
| - Institute actions to establish and support breathing and oxygenation, refer for continuing treatment as needed | 8.1 | 64.5 | 53.7 |
| - Provide a safe warm environment for initiating breastfeeding and attachment (bonding) in the first hour of life | 75.6 | 75.8 | 58.0 |
| - Conduct a complete physical examination of new-born in presence of mother/family; explain findings and expected changes e.g., color of extremities, moulding of head, and refer for abnormal findings | 25.6 | 69.6 | 56.8 |
| - Institute new-born prophylaxis e.g., ophthalmic infection, and other diseases, according to policies and guidelines | 23.3 | 59.9 | 53.1 |
| - Promote care by mother, frequent feeding and close observation | 39.5 | 72.9 | 64.3 |
| - Involve partner/support persons in providing new-born care | 53.5 | 73.2 | 68.2 |
