# Supplementary material for: Validating midwifery professionals’ scope of practice and competency: A multi-country study comparing national data to international standards
Source: PLoS One. 2023 May 25;18(5):e0286310. doi: 10.1371/journal.pone.0286310 (PMC10212099; doi:10.1371/journal.pone.0286310)
Supplement: S3 Table — (DOCX) [file pone.0286310.s003.docx]

**S3_Table**

**Table 3C:** All Skill Assessment of ICM Behaviours for **Midwives in** Category 4: Ongoing Care of Women and Newborns

|  | **Argentina** | **Ghana** | **India** |
| --- | --- | --- | --- |
| Total number of midwives | **86** | **414** | **766** |
|  | % | % | % |
| **4.a Provide postnatal care for the healthy woman** |  |  |  |
| - Review history of pregnancy, labour, and birth | 77.9 | 73.7 | 49.6 |
| - Conduct a focused physical exam to assess breast changes and involution | 84.9 | 70.0 | 49.0 |
| - Monitor blood loss and other body functions | 83.7 | 62.1 | 42.6 |
| - Assess mood and feelings about motherhood and demands of infant care | 54.7 | 69.6 | 35.3 |
| - Provide pain control strategies if needed for uterine contractions, and perineal trauma | 52.3 | 63.5 | 39.4 |
| - Provide information about self-care that enables mother to meet needs of new-born, e.g., adequate food, nutritional supplements, usual activities, rest periods, and household help | 54.7 | 73.7 | 55.2 |
| - Provide information about safe sex, family planning methods appropriate for the immediate postnatal period, and pregnancy spacing | 77.9 | 79.5 | 65.0 |
| **4.b Provide care to healthy new-born infant** |  |  |  |
| - Examine infant at frequent intervals to monitor growth and developmental behaviour | 7.0 | 76.6 | 50.4 |
| - Distinguish normal variation in new-born appearance and behaviour from those indicating pathologic conditions | 8.1 | 69.8 | 44.5 |
| - Administer immunizations, carry out screening tests as indicated | 9.3 | 74.4 | 47.3 |
| - Provide information to parents about a safe environment for infant, frequent feeding, care of umbilical cord, voiding and stooling, and close physical contact | 36.1 | 80.2 | 71.8 |
| **4.c Promote and support breastfeeding** |  |  |  |
| - Promote early and exclusive breastfeeding while respecting a woman’s choice regarding new-born feeding | 70.9 | 83.6 | 74.2 |
| - Provide information about infant needs, frequency and duration of feedings, and weight gain | 62.8 | 81.9 | 69.5 |
| - Provide support and information about breastfeeding for a minimum of six months, including combining with work, maintaining milk supply, and storing breast milk | 59.3 | 81.6 | 64.6 |
| - Identify and manage breastfeeding problems (e.g. mastitis, low milk supply, engorgement, improper latch) | 51.2 | 79.2 | 69.2 |
| - Provide information to women breastfeeding multiple new-borns | 34.9 | 80.2 | 67.4 |
| - Refer women to breastfeeding support as indicated | 51.2 | 79.5 | 64.5 |
| - Advocate for breastfeeding in family and community | 57.0 | 80.0 | 60.1 |
| **4.d Detect, treat, and stabilise postnatal complications in woman and refer as necessary** |  |  |  |
| - Provide information to woman and family about potential complications and when to seek help | 59.3 | 71.0 | 33.0 |
| - Assess woman during postnatal period to detect signs and symptoms of complications | 67.4 | 70.8 | 31.5 |
| - Distinguish postnatal depression from transient anxiety about caring for baby, assess availability of help and support at home, and provide emotional support | 24.4 | 66.4 | 32.9 |
| - Provide counselling and follow-up care for women and family members who experience stillbirth, neonatal death, serious infant illness, and congenital conditions | 8.1 | 68.6 | 44.7 |
| - Provide first line measures to treat or stabilize identified conditions | 27.9 | 65.5 | 32.6 |
| - Arrange referral and/or transfer as needed | 44.2 | 70.8 | 52.6 |
| **4.e Detect, stabilise, and manage health problems in new-born infant and refer if necessary** |  |  |  |
| - Provide information to woman and family about potential complications and when to seek help | 25.6 | 72.0 | 38.1 |
| - Assess new-born infant during postnatal period to detect signs and symptoms of complications | 7.0 | 71.3 | 37.1 |
| - Distinguish postnatal depression from transient anxiety about caring for baby, assess availability of help and support at home, and provide emotional support | 15.1 | 64.5 | 36.3 |
| - Provide counselling and follow-up care for women and family members who experience stillbirth, neonatal death, serious infant illness, and congenital conditions | 8.1 | 68.1 | 38.6 |
| - Provide first line measures to treat or stabilize identified conditions | 4.7 | 64.7 | 37.5 |
| - Arrange referral and/or transfer as needed | 9.3 | 69.8 | 50.4 |
| **4.f Provide family planning services** |  |  |  |
| - Provide and protect privacy and confidentiality for discussions about family planning knowledge, goals for limiting and/or spacing of children, and concerns and myths about methods | 74.4 | 83.6 | 63.7 |
| - Obtain relevant history of use of methods, medical conditions, sociocultural values, and preferences that influence choice of method | 61.6 | 80.2 | 64.4 |
| - Provide information about how to use, effectiveness, and cost of various methods to support informed decision-making | 64.0 | 78.7 | 64.1 |
| - Provide methods according to scope of practice and protocols, or refer to another provider | 79.1 | 77.8 | 60.7 |
| - Provide follow-up assessment of use, satisfaction, and side-effects | 55.8 | 76.1 | 53.4 |
| - Refer for woman or partner for sterilization procedure | 55.8 | 71.7 | 61.1 |
